# The Brighter Side of Mental Hospital Life

**Published:** 1913-08-23

**Authors:** 


### THE BRIGHTER SIDE OF MENTAL HOSPITAL LIFE.

### Patients' Tastes and Faculties for Amusement.

If there is one aspect of life in a civilised community which provides no scope for the *laudator temporis acti*, it is when mental hospitals form the subject of discussion. There may have been good old days—or perchance the contrast might be made clearer by speaking of them as better old days—in respect of certain ways of life, but it would certainly not be true if asylums form the topic. In respect of them it may be claimed that the last few decades have witnessed enormous changes for a better and more humane method of treatment.

So much, however, has been written of the sombre side of this life that even now the public is inclined to believe that it is almost entirely a period of gloom, unrelieved by any news of cheerfulness, and characterised by a necessary abandonment of hope by all those who enter into it. Charles Reade and many others since his time have undoubtedly contributed greatly towards the strength of this impression; and there has been of late a marked recrudescence of this form of literary venture. Unfortunately, too, certain members of the medical profession have lent the weight of their names—not always exhibiting a corresponding accuracy of information—to statements which, by giving rise to such feelings as to mental hospital life in the minds of the relatives of those whom the exigencies of society send to a necessary segregation, cause infinite pain and distress. More than this, so strong at times is this feeling, that many patients are withheld from skilled care and attention until the possibility of cure has almost or entirely passed away.

Whilst no one who is cognisant of the actual state of affairs in a mental hospital will deny that there is much sadness and vexation of spirit to be found in such institutions, nevertheless it is well known that there is quite a different picture, and one of a much brighter hue. It is amazing how many of those who write about mental hospitals seem unaware of the varied amusements which help to make the time pass more easily, and which serve at the same time as the most useful adjuncts in restoring certain of the patients to mental health. To name some of these, there are dancing, concerts, garden-parties, cricket matches, cinematograph entertainments, dramatic performances, fancy-dress balls, and, in addition, whist, bridge, chess, draughts, billiards; of course, there are other means of alleviating the necessary restriction of liberty, but in the meantime let these suffice to show that the patients do not spend their time despondently ruminating on their hard fates or madly assaulting those in their proximity.

Dancing is, in the winter time at least, probably the most popular form of amusement, and the dances are looked forward to with keen anticipation. And if it should be decided that fancy dress is to be worn, there will be days of excitement, wherein the details of the proposed costumes will be discussed, this suggestion being negatived, that finally accepted. The workrooms are laid under tribute for oddments and superfluities, and these are transformed, often with no mean skill, into costumes which even a professional eye may scan with admiration. There is much mutual assistance, too, among the patients, not only in suggesting schemes, but also in the actual workmanship; for, though altruism is not too prominent a feature among the insane, there are many who will grudge neither time nor trouble in assisting their neighbours. Then comes the night of the dance and all the surprises which it brings, for many of the costumes have been prepared with great secrecy in order that their novelty may be the more striking when the time for displaying them arrives. The dancing itself is indulged in with zest, and on these occasions, of course, the sexes intermingle, and this with a propriety and with a due observance of conventional usages which would do no discredit to any well-conducted evening party. True, there are occasions when the excitement may prove too great for certain individuals, who will have to leave ere the dance is finished; this is, however, fortunately rare. The enjoyment is apparent, and the effects mentally and physically are excellent, so that dancing, quite apart from any hedonistic point of view, is a valuable adjunct to treatment in psychiatric practice.

Concerts, too, are much appreciated and greatly enjoyed—if good. It is necessary to emphasise the desideratum of such performances being of high quality, because there seems to be an impression among performers that anything is good enough for the insane. Nothing could be further from the truth, as one impresario found who visited a mental hospital. His performers had given a very poor exhibition of their powers, and they were received in a rather chilly fashion. After the performance was over he remarked to me, "Your people here don't seem very enthusiastic!" to which I had to answer that they did not: politeness forbade me from adding, "On this particular occasion," though the addendum would have been eminently justified. It is no use to endeavour to foist mediocre performances upon the insane; they are severely critical, and will on occasion give vent to their criticisms with no uncertain voice. But, on the other hand, should the performance be worthy of commendation there will be no lack of enthusiastic applause, whilst humorous items and incidents will not be received with any greater absence of appreciation than they would be by the average concert or theatre audience.

Outdoor and indoor games are as a rule played with interest, frequently with skill, and almost invariably with a restraint which is not too often witnessed outside a mental hospital. Many are the hard-fought chess-matches, and draughts has often notable exponents who, though at other times they may exhibit marked morbid mental symptoms, evince a knowledge of the moves which would put to rout many sane opponents, thus showing how mental divagations may be kept completely in abeyance by the concentration of the mind upon pursuits which demand strenuous intellectual effort. Whist and bridge have their devotees, who will spend many hours in the pleasant rivalry such games entail; and at intervals whist drives are arranged in which patients of both sexes take part, while the evening is usually brought to a conclusion with music, singing, and reciting.

These are, however, so to speak, the more or less public forms of entertainment, and do not furnish, after all, the best criterion for judging of the real and intimate life of the establishment. But here also can be observed much that will lead to the formation of an optimistic opinion. How are these other times passed? By some truly in noisiness and bickering, in black despondency, and in petulant complaining; let the picture not be painted in too rosy colours, nor in a vague impressionistic sketch, let the background be so ill-defined that no clear conception can be obtained. For such things do exist in the best mental hospitals, and will, in the natural order of things, always exist so far as the limits of the human intelligence can foresee. Granted, however, for the sake of right perspective that such undesirable traits are to be found, yet it can be argued with much force that they are not present in the overwhelming degree some would have us believe. Instead—or rather alongside these—we may see keen appreciation of the simple pleasures of life and an interest in current events rendered more intense by detachment from participation in them. There is fancy-work to be done, and a newcomer shows how a different stitch may be employed or some other pattern may be copied; a new game of patience has to be explained and learned; someone has a puzzle to be solved which needs suggestions from all and sundry; yet another has been out with a friend to a theatre and is expected to describe the performance to an interested circle; and so the time passes. A never-failing source of pleasure is, it is hardly necessary to state, music; and the piano is naturally the instrument most in use. Many of the patients are skilled performers, and so with playing and with singing many an evening passes rapidly and pleasantly away. The range of choice is catholic in the extreme; a Beethoven Sonata will be observed keeping company with one of Harry Lauder's ditties; while Grieg and Brahms may give place to Wilkie Bard and R. G. Knowles! It is curious to observe, too, how the power of musical exposition may survive the comparative ruin of the intellectual faculties; so that a person who cannot remember what he ate at his latest meal or is unable to converse intelligibly, will yet be capable of playing difficult and complex musical compositions.

It will almost go without saying that reading is one of the most popular methods of passing the time, and that books form the greatest solace for many of the inhabitants. Nor is the reading confined by any means to works of fiction; though, of course, in mental hospitals, as elsewhere, they are in greatest demand. On the other hand volumes of reminiscences, biographies, and books on travel have many readers. One patient varies the study of Anglo-Saxon with learning Dutch, and passes from this again to reading about the language of the Hittites; another is devoted to travel—Sven Hedin, Peary, Prejevalsky, the Abbé Huc; a third is found absorbed in Nordau, Benson's essays, or the novels of H. G. Wells. Scott, Thackeray, Dickens, Lytton, and more modern writers such as W. J. Locke, Maurice Hewlett, Conan Doyle, Marion Crawford, all have their appreciative admirers.

Apropos of books and reading, I recall one patient of extremely interesting personality. In addition to a devouring love for literature he had the fortunate asset of a retentive memory. He would sit for hours and pour out his reflections on things in general—on men, on events, and, above all, on books. La Rochefoucauld probably never had a more fervent admirer, and he would wax eloquent over the pithy aphorisms of that satirical aristocrat. Anatole France he lauded and loved; Alphonse Daudet, the "French Dickens," was a bosom friend. From Byron he would quote by the page; Shelley rolled sonorously forth from his lips, the sensuous beauty of some of the passages being imbued with a rhetorical fervour rarely found even in the professed reciter. Once I lent him Wilde's "The Canterville Ghost," and I cannot recall any such enthusiastic admiration as he expressed. For days he would ask those whom he met, "Have you read 'The Canterville Ghost?' Oh, the beauty of it! The lovely thoughts!" I know that for a time it took the place of all other literature in his mind; and truly it is a wonderfully dainty piece of artistry, and may well be likened to an elegant and clear-cut cameo. Alas, poor Wilde, there were more points of resemblance between him and some of the inmates of mental hospitals than ever certain of the ignorant, the prejudiced and, perchance, the pharisaical, would admit. But I have let my old friend and his perfervid admiration of Wilde lead me away from the subject in hand.

I hope I have shown, in howsoever inefficient a manner, that there is another side of mental hospital life from the one usually depicted: one where there is something else than tribulation and agony of spirit. And this is true, that I have seen patients who, a return to mental health having necessitated their departure from the hospital circle, have wept, because, by leaving, ties of affection and of friendship were sundered; others who will return again and again of their own free will when they feel that presently their nervous organisation will succumb to the shocks and stresses of everyday life, and who seek while there is yet time the protection of those stone walls which do not make for them a prison. And some there are who could tell you—as they have told me—that some of the happiest days of their lives have been passed within an asylum. I trust that the inference is plain!

